# Predicting the prognosis of Wilms tumor by peripheral blood cells: a real-world study of more than 30 years

**DOI:** 10.1186/s13052-024-01805-8

**Published:** 2024-11-14

**Authors:** Lin Jie, Shi Qin-Lin, Tian Xiao-Mao, Hong Peng, Wang Zhuang-Cheng, Hu Zai-Hong, Cui Kong-Kong, Gao Zhi-Qiang, Liu Feng, Lin Tao, Wei Guang-Hui

**Affiliations:** 1 Department of Urology Children’s Hospital of Chongqing Medical University. National Clinical Research Center for Child Health and Disorders, Ministry of Education Key Laboratory of Child Development and Disorders, Chongqing Key Laboratory of Structural Birth Defect and Reconstruction, Children’s Hospital of Chongqing Medical University, Chongqing, 400014 China; 2 Department of Pediatric Urology, Key Laboratory of Birth Defects and Related Diseases of Women and Children (Sichuan University), Ministry of Education, West China Second University Hospital, Sichuan University, Chengdu, 610000 China

**Keywords:** Wilms tumor, Blood cells, Overall survival, Risk stratification, Prognosis

## Abstract

**Background:**

Despite established excellent treatment strategies for Wilms tumor (WT), effective prognostic evaluation methods were lacking. This study aims to examine prognostic factors for WT through real-world peripheral blood cell profiling.

**Methods:**

Basic data and pre-treatment laboratory indices from WT and non-WT children underwent Wilcoxon test analysis. Chi-square tests assessed the correlation between blood cells and the overall survival (OS) and event-free survival (EFS) of WT. Further the Log-rank test and multivariate Cox were used to identify independent prognostic factors for OS. Traditional accepted factors were included in multi-Cox and the nomogram was constructed to further validate the outcome.

**Results:**

Blood cells significantly differed between WT and non-WT groups (*P* < 0.05). Univariate analysis revealed that NLR above 1.380, stage IV, M below 0.325 × 10^3^/μL were linked with lower OS, and PLR below 94.632, LB above 3.570 × 10^3^/μL, stage IV, M above 0.325 × 10^3^/μL,age ≤ 3 years were meaningful for higher EFS (*P* < 0.05). While in the multifactorial COX, only M (HR:0.220, HR95%CI: 0.080 ~ 0.620, *P* = 0.004 and HR: 0.437, HR95%CI: 0.202 ~ 0.947, *P* = 0.036, respectively) and stage IV (HR: 7.890, HR95%CI: 1.650 ~ 37.770, *P* = 0.010 and HR: 3.720, HR95%CI: 1.330 ~ 10.408, *P* = 0.012, respectively) were independent prognostic factors for OS and EFS. These two variables also were significant after including recognized risk factors, and were demonstrated the predictability via nomogram.

**Conclusions:**

OS and EFS were poorer in WT children with M below 0.325 × 10^3^/μL, suggesting the potential as a prognostic predictor for WT.

**Supplementary Information:**

The online version contains supplementary material available at 10.1186/s13052-024-01805-8.

## Introduction

Wilms tumor (WT) was the most common embryonic tumor in pediatric urology, with a majority of cases diagnosed in children under 5 years of age. It constitutes approximately 90% of childhood malignant renal tumors [1]. Upon diagnosis, WT patients typically remain asymptomatic, and only about 35% of cases present with symptoms such as hematuria, hypertension, or flank pain [2]. Over the past 50 years, there have been significant improvements in the treatment outcomes for Wilms tumor patients, resulting in an overall survival (OS) rate exceeding 90% [3]. However, for children experiencing recurrence, metastasis, cachexia, or displaying insensitivity to radiotherapy and chemotherapy, the treatment outcomes are still unsatisfactory [4].

There is an increasing focus among experts on the need to develop an alarm system for predicting potential serious problems. Peripheral blood cells, being readily accessible indicators, have shown significant correlations with tumorigenesis and disease progression. Research has demonstrated that blood cells can exert anti-tumor effects through direct killing, antigen presentation, and secretion of inflammatory mediators, or pro-tumor effects by promoting angiogenesis and inducing gene mutations [5]. Moreover, the neutrophil–lymphocyte ratio (NLR) in peripheral blood has proven to be a useful marker for prognostic assessment and treatment response in adult cervical cancer [6], ovarian cancer [7], and pediatric osteosarcoma [8]. Monocytes in peripheral blood have also been identified as prognostic predictors in adult hepatocellular cancer [9], esophageal [10] cancers, and lymphoma [11]. Nevertheless, in the case of Wilms tumor, although studies have shown correlations between C-reactive protein (CRP), lymphocyte-to-monocyte ratio (LMR), and prognosis [12], there is still a lack of simple, systematic, real-world data analysis based on blood cells.

In this study, we included pre-treatment peripheral blood cells based on previous real data, and assessed the prognosis of the tumor according to data models in recent years, such as chi-square test, COX regression, and especially nomogram.

## Method

### Data collection and selection standard

**Fig. 1:**
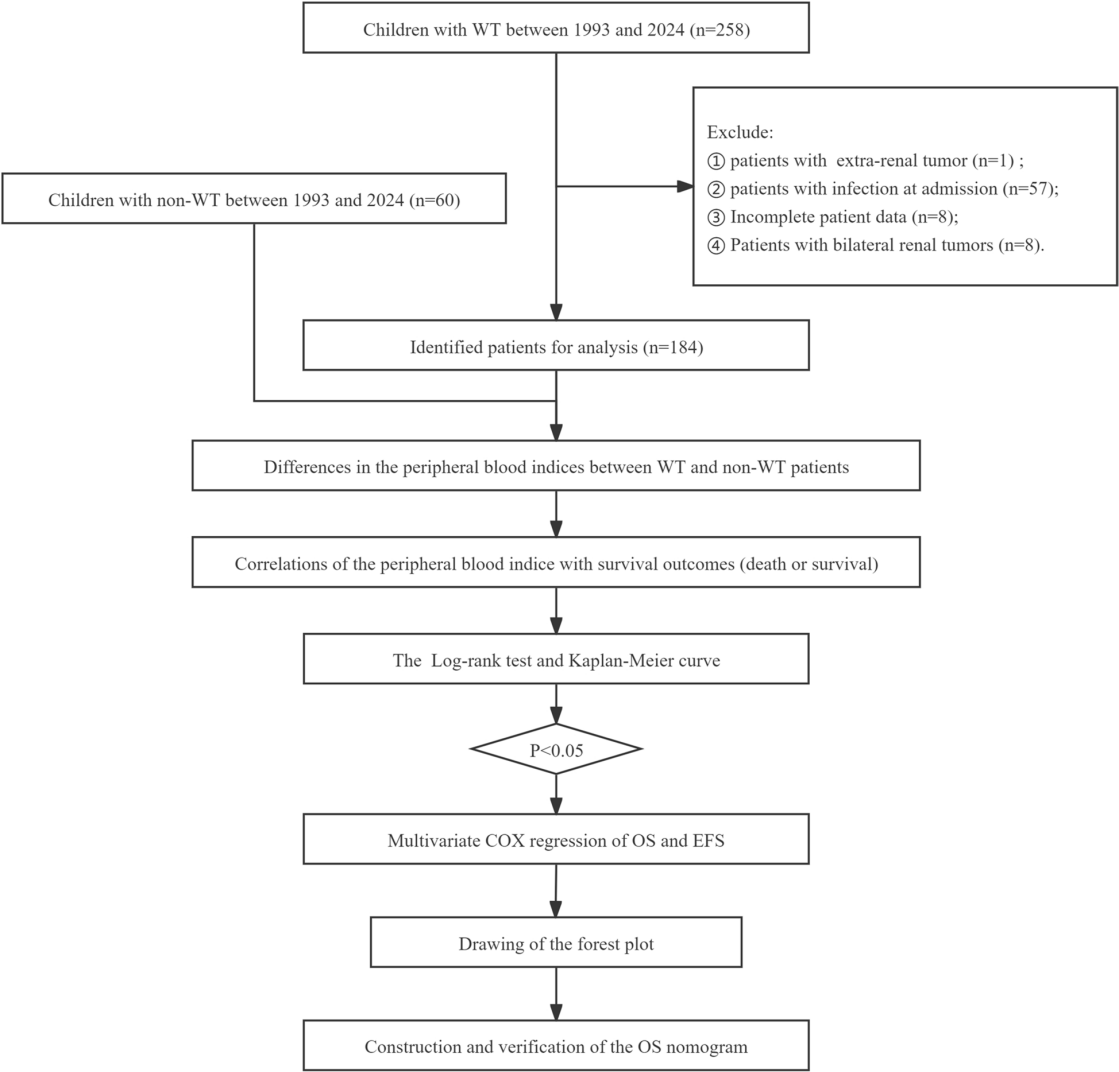
The flowchart of patient screening and experimental design. WT Wilms tumor, OS overall survival rate

We extracted clinical and pathological data of children with WT and non-WT from the Children’s Hospital of Chongqing Medical University between March 1993 and March 2024. The specific process of patient screening and study design can be found in Fig. 1. The collected data included demographics (age and gender at diagnosis) of WT and non-WT children, pathological reports (pathological type), Tumor clinical features (tumor laterality, postoperative tumor stage), peripheral blood cells of patients with WT and non-WT at admission which included in white blood cell (WBC), red blood cell (RBC), platelet count (PLT), hemoglobin count (HB), absolute lymphocyte count (LB), absolute neutrophil count (N), absolute monocyte count (M) and lymphocyte percentage (LB%), neutrophil percentage (N%), monocyte percentage (M%), and calculation of platelet-lymphocyte ratio (PLR) and neutrophil -lymphocyte ratio (NLR), and follow-up findings (death, recurrence status, metastasis status). This study was carried out according to the Declaration of Helsinki and approved by the Ethics Committee of the Children’s Hospital of Chongqing Medical University. The retrospective nature of the study exempted the requirement for informed consent.

Follow-up information was collected through various methods, including physical and online outpatient follow-up, We Chat consultations, telephone interviews, and review of medical histories. Pathological information was obtained from the Department of Pathology Children`s Hospital of Chongqing Medical University. Postoperative stage (stage I-IV) was determined based on the COG protocol. The overall survival (OS) was defined as the time from admission to death from any cause or last follow-up, while event-free survival (EFS) was defined as the time from admission to first disease recurrence, metastasis, death from any cause, or last follow-up. Due to the limited number of cases, bilateral patients were excluded when considering tumor laterality.

A total of 184 patients with WT were included in the study: ① patients aged < 18 years; ② patients who were pathologically diagnosed with WT after surgery; ③ patients who did not have any history of tumor-related treatment past or now; and ④ Patients who provided complete demographic, clinicopathologic, and follow-up information.

The exclusion criteria were as follows: ① patients with extra-renal WT or no WT (including renal clear cell sarcoma, renal cell sarcoma, malignant rhabdoid tumor of kidney, etc.); ② patients with infection at admission; ③ patients without pathological diagnosis; and ④ patients with bilateral renal tumors.

Thirty males with syringomyelia and 30 females with nacreous cysts were enrolled in the non-WT group. The criteria for the selection were: ① patients with no infection and other comorbidities at admission; ② Patients with surgically confirmed syringomyelia or nacreous cysts.

### Analysis of prognostic factors

In this study, the differences in peripheral blood indices between Wilms tumor (WT) and non-WT patients were analyzed using the Wilcoxon rank test. Additionally, the blood cells of WT patients were divided into two groups based on the median, and the correlations of these blood cells with survival outcomes, including overall survival (OS) and event-free survival (EFS), were analyzed using the Chi-square test. For the survival analysis, the Kaplan–Meier method and log-rank test were employed to compare the differences between the groups. Data with a significance level of *P* < 0.05 were included in the multivariate Cox regression analysis to identify independent prognostic factors for OS and EFS and a forest plot was then drawn to visualize the results. To further validate the findings, the conventional universally accepted prognostic factors for WT were included in the multivariate Cox regression analysis. The recognized risk factors were defined type and stage. (In our study, only stage and type were enrolled. As for the other clinical and biologic indexes which were used by SIOP and COG to develop the treatment approaches [13], including tumor volume, age, loss of heterozygosity (LOH) at chromosomes 1 p and 16 q and response to chemotherapy, were all excluded. This is because these indicators exclusively apply to a small subgroup of patients. For instance, LOH at 1 p/16 q is a negative prognostic marker for recurrence of WT with favorable histology. However, it occurs in approximately 5% of patients [14].) Subsequently, the 184 patients were randomly assigned to a training cohort and a validation cohort, with a split ratio of 7:3. In the training cohort, univariate Cox analysis was performed, and variables were selected for multivariate Cox analysis using bidirectional stepwise regression. Based on the independent risk factors obtained from the Cox regression analysis, a nomogram was constructed for the prediction of 3-year, 5-year, and 8-year OS rates in WT patients. In order to assess the discriminative power, we plotted the receiver operating characteristic curve (ROC), and calculated the area under the ROC curve (AUC) and the consistency index (C-index). Calibration curves were constructed for 500 resamples using bootstrap method to compare the compatibility of predicted OS with the actual OS. At the same time, the decision curve analysis (DCA) was depicted to show the net benefit of different models.

### Statistical analyses

The clinicopathological characteristics were presented as n (%) and the continuous variable data were expressed as median (25th percentile, 75th percentile). The results of COX regression reported as hazard ratio (HR) with 95% confidence intervals (95%CI). A two-side *P* < 0.05 was considered statistically significant. Data processing, analysis and figures plotting were performed using R version 4.3.0, Zstats (www.medsta.cn/software), along with GraphPad Prism 9.5.1.

## Results

### Characteristics of patients

Following screening, a total of 184 patients with WT were included in this study and the base line data were shown in Table 1. 184 Patients were found to have 162 (88.04%) favorable histology (FH), 99 (53.80%) females and 85 (46.20%) males, 88 (47.83%) left and 96 (52.17%) right. There were 33 patients with adverse events: 10 (5.43%) recurrence, 19 (10.33%) death, and 14 (7.61%) metastases which include 9 (4.89%) pulmonary, 1 (0.54%) hepatic, 1 (0.54%) intestinal, 1 (0.54%) lung plus thoracic with mediastinal, 1 (0.54%) pelvic plus hepatic and 1 (0.54%) thoracic plus abdominal metastases).

**Table 1:** Characteristics of patients with WT

Characteristics								n(%)		
Age										
		≤ 3Years						112(60.87)		
		> 3Years						72(39.13)		
Gender										
		Males						85(46.20)		
		Females						99(53.80)		
Stage										
		I						41(22.28)		
		II						53(28.80)		
		III						61(33.15)		
		IV						29(15.76)		
Type										
		FH						162(88.04)		
		uFH						22(11.96)		
Laterality										
		Left						88(47.83)		
		Right						96(52.17)		
Outcome										
		Recurrence						10(5.43)		
		Death						19(10.33)		
		Metastases						14(7.61)		
		Pulmonary metastases						9(4.89)		
		Hepatic metastases						1(0.54)		
		Lung,chest and mediastinal metastases						1(0.54)		
		Intestinal metastasis						1(0.54)		
		Pelvic and hepatic metastases						1(0.54)		
		Thoracic and abdominal metastases						1(0.54)		

*n* number, *FH* favorable histology, *uFH* unfavorable histology

### Analysis of prognostic factors in WT

#### Peripheral blood cells compared between patients with WT and non-WT children

**Fig. 2:**
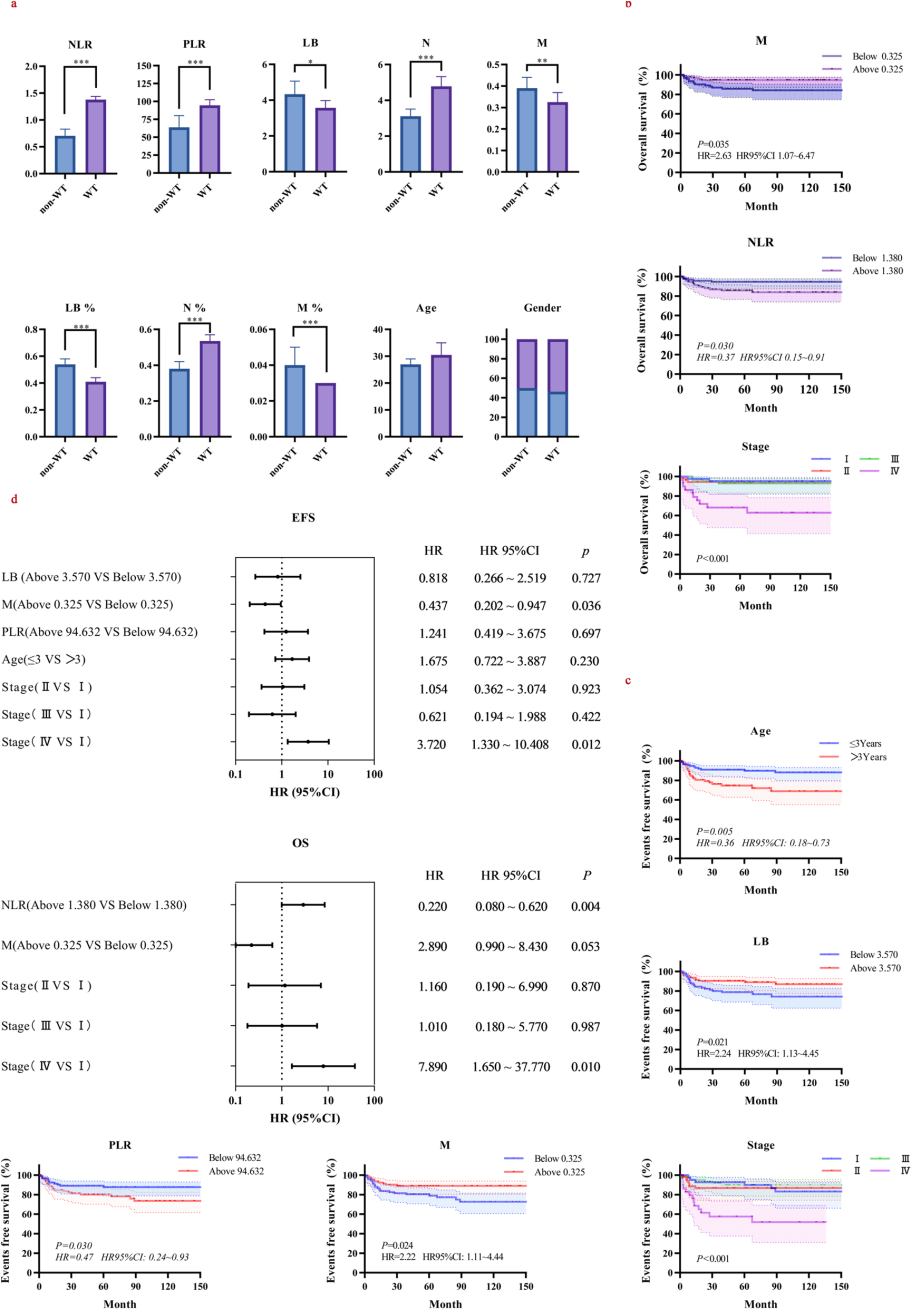
The Wilcoxon rank test and survival analysis. **a** Histogram of the analysis of variance for WT and non-WT children; **b **The Kaplan–Meier curve for overall survival rate in WT patients; **c **The Kaplan–Meier curve for event-free survival rate in WT patients; **d **Forest graphs of OS and EFS in WT patients. * *P* < 0.5, ** *P* < 0.01, *** *P* < 0.001, WBC white blood cell count, PLT platelet count, RBC red blood cell count, HB hemoglobin, LB absolute lymphocyte count, N absolute neutrophil count, M absolute monocyte count, LB% lymphocyte percentage, N% neutrophil percentage, M% monocyte percentage, PLR platelet-lymphocyte ratio, NLR neutrophil–lymphocyte ratio, *P* *P*-value, n number, OS overall survival, EFS event-free survival

To investigate potential differences in blood cells of the children with WT and non-WT, we carried out the Wilcoxon rank test. The results showed that there were no significant differences in age and gender between the two groups (*P* > 0.05). However, there were notable variations in various blood cell parameters. WBC, PLT, N and N% in WT patients were higher than non-WT individuals, while HB, RBC, LB, LB%, M, M% were lower (*P* < 0.05). (As in Fig. 2a, we only show the key information in the main text, and detailed information can be found in Table S1 of the additional file.)

#### Analysis of the correlation between peripheral blood cells and tumor survival outcomes

Log-rank test was performed to compare blood indexes and survival outcomes (death or survival, event-free and positive events). As shown in Table 2, Monocyte count (M) below 0.325 × 10^3^/μL (*P* = 0.029) and NLR above 1.380 (*P* = 0.029) accounted for more of the patients who died. Patients who had positive events presented a higher percentage of PLR above 94.632 (*P* = 0.035), LB below 3.570 × 10^3^/μL (*P* = 0.029) and M below 0.325 (*P* = 0.035) × 10^3^/μL than those with event free.

**Table 2:** Correlation analysis of pretreatment peripheral blood cells and tumor survival outcomes

Variables		OS		*P*	EFS		*P*
		Survival	Death		Event free	Positive events	
PLR	Below 94.632	86(52.12)	6(31.58)	0.090	81 (53.64)	11 (33.33)	0.035*
	Above 94.632	79(47.88)	13(68.42)		70 (46.36)	22 (66.67)	
NLR	Below 1.380	87(52.73)	5(26.32)	0.029*	79 (52.32)	13 (39.39)	0.179
	Above 1.380	78(47.27)	14(73.68)		72 (47.68)	20 (60.61)	
WBC (× 10^3^/μL)	Below 9.270	81(49.09)	11(57.89)	0.467	74 (49.01)	18 (54.55)	0.564
	Above 9.270	84(50.91)	8(42.11)		77 (50.99)	15 (45.45)	
PLT (× 10^3^/μL)	Below 326.500	81(49.09)	11(57.89)	0.467	73 (48.34)	19 (57.58)	0.337
	Above 326.500	84(50.91)	8(42.11)		78 (51.66)	14 (42.42)	
RBC (× 10^6^/μL)	Below 4.270	80(48.48)	10(52.63)	0.732	75 (49.67)	15 (45.45)	0.661
	Above 4.270	85(51.52)	9(47.37)		76 (50.33)	18 (54.55)	
HB (g/L)	Below 108.000	77(46.67)	9(47.37)	0.954	73 (48.34)	13 (39.39)	0.351
	Above 108.000	88(53.33)	10(52.63)		78 (51.66)	20 (60.61)	
LB (× 10^3^/μL)	Below 3.570	78(47.27)	13(68.42)	0.081	69 (45.70)	22 (66.67)	0.029*
	Above 3.570	87(52.73)	6(31.58)		82 (54.30)	11 (33.33)	
N (× 10^3^/μL)	Below 4.780	84(50.91)	8(42.11)	0.467	77 (50.99)	15 (45.45)	0.564
	Above 4.780	81(49.09)	11(57.89)		74 (49.01)	18 (54.55)	
M (× 10^3^/μL)	Below 0.325	78(47.27)	14(73.68)	0.029*	70 (46.36)	22 (66.67)	0.035*
	Above 0.325	87(52.73)	5(26.32)		81 (53.64)	11 (33.33)	
LB%	Below 0.410	78(47.27)	13(68.42)	0.081	71 (47.02)	20 (60.61)	0.157
	Above 0.410	87(52.73)	6(31.58)		80 (52.98)	13 (39.39)	
N%	Below 0.535	85(51.52)	7(36.84)	0.226	78 (51.66)	14 (42.42)	0.337
	Above 0.535	80(48.48)	12(63.16)		73 (48.34)	19 (57.58)	
M%	Below 0.030	37(22.42)	7(36.84)	0.163	36 (23.84)	8 (24.24)	0.961
	Above 0.030	128(77.58)	12(63.16)		115 (76.16)	25 (75.76)	

Data are presented as n(%)

*WBC* white blood cell count, *PLT* platelet count, *RBC* red blood cell count, *HB* hemoglobin, *LB* absolute lymphocyte count, *N* absolute neutrophil count, *M* absolute monocyte count, *LB%* lymphocyte percentage, *N%* neutrophil percentage, *M%* monocyte percentage, *PLR* platelet-lymphocyte ratio, *NLR* neutrophil–lymphocyte ratio, *P* *P*-value, *n* number, *OS* overall survival, *EFS* event-free survival

*P < 0.5

#### The Kaplan–Meier survival curves of peripheral blood cells before treatment

The Kaplan–Meier survival curves for blood cells are displayed in Fig. 2b-c. (In the main text, we present only significant results; additional information is available in Fig. S1-S2 in the additional file.) In the entire study population, there were 19 (10.33%) deaths, with a 5-year OS of 90.22% and a 5-year EFS of 83.15%. Factors demonstrating significantly inferior overall survival by the log-rank test were NLR above 1.380 (HR 0.37, HR95% CI 0.15 ~ 0.91, *P* = 0.030), stage IV (*P* < 0.001) and M below 0.325 × 10^3^/μL (HR 2.63, HR95% CI 1.07 ~ 6.47, *P* = 0.035). Simultaneously, higher EFS was associated with stage IV (*P* < 0.001), Age ≤ 3 years (HR 0.36, HR95% CI 0.18 ~ 0.73, *P* = 0.005), PLR below 94.632 (HR 0.47, HR95% CI 0.24 ~ 0.93, *P* = 0.030), LB above 3.570 × 10^3^/μL (HR 2.24, HR95% CI 1.13 ~ 4.45, *P* = 0.021) and M above 0.325 × 10^3^/μL (HR 2.22, HR95% CI 1.11 ~ 4.44, *P* = 0.024).

#### Multivariate cox regression analysis of prognostic factors correlated with WT

Variables with *P* < 0.05 in log-rank test were included in the multifactor COX analysis, and forest plots were developed (see Fig. 2d. The corresponding tables are available in Table S2-S3 in the additional file). A total of 2 factors were independently correlated with OS and EFS: M (HR 0.22, HR95%CI 0.08 ~ 0.62, *P* = 0.004 and HR 0.44, HR95%CI 0.20 ~ 0.95, *P* = 0.036, respectively) and stage IV (HR 7.89, HR95% CI 1.65 ~ 37.77, *P* = 0.010 and HR 3.72, HR95% CI 1.33 ~ 10.41, *P* = 0.012).

### Multivariate cox regression after including conventional factors

To exclude the effect of traditional accepted risk factors, we put these factors with significant variables in log-rank test into multi-COX. As shown in Tables 3– 4, both M (HR 0.23, HR95%CI 0.08 ~ 0.66, *P* = 0.006 and HR 0.43, HR95%CI 0.20~0.91, *P*=0.026, respectively) and Stage IV (HR 7.12, HR95%CI 1.48 ~ 34.22, *P* = 0.014 and HR 3.79, HR95%CI 1.37 ~ 10.44, *P* = 0.010, respectively)were independently associated with OS and EFS of WT.

**Table 3:** Multivariate Cox regression of OS in WT after including conventional factors

Variables		HR	95%CI	*P*
NLR				
	Below 1.380	Ref		
	Above 1.380	2.89	0.97 ~ 8.61	0.057
M (× 10^3^/μL)				
	Below 0.325	Ref		
	Above 0.325	0.23	0.08 ~ 0.66	0.006**
Stage				
	I	Ref		
	II	1.17	0.19 ~ 7.04	0.864
	III	0.92	0.16 ~ 5.50	0.930
	IV	7.12	1.48 ~ 34.22	0.014*
Type				
	FH	Ref		
	uFH	1.68	0.46 ~ 6.19	0.433

*M* absolute monocyte count, *NLR* neutrophil–lymphocyte ratio, *P P*-value, *n* number, *FH* favorablehistologdency, *uFH* unfavorable histology, *Ref* reference, *HR *Hazard Ratio, *CI *Confie Interval

^*^P < 0.5, **P < 0.01

**Table 4:** Multivariate Cox regression of EFS in WT after including conventional factors

Variables		HR	95%CI	*P*
PLR				
	Below 94.632	Ref		
	Above 94.632	1.40	0.50 ~ 3.90	0.525
LB (× 10^3^/μL)				
	Below 3.570	Ref		
	Above 3.570	0.74	0.26 ~ 2.12	0.578
M (× 10^3^/μL)				
	Below 0.325	Ref		
	Above 0.325	0.43	0.20 ~ 0.91	0.026*
Stage				
	I	Ref		
	II	1.08	0.37 ~ 3.13	0.888
	III	0.60	0.19 ~ 1.94	0.392
	IV	3.79	1.37 ~ 10.44	0.010*
Type				
	FH	Ref		
	uFH	1.43	0.53 ~ 3.90	0.483

*LB* absolute lymphocyte count, *LB* absolute lymphocyte count, *M* absolute monocyte count, *NLR* neutrophil–lymphocyte ratio, *P* *P*-value, *n* number, *FH* favorablehistologdency, *uFH* unfavorable histology, *Ref* reference, *HR* Hazard Ratio, *CI* Confie Interval

^*^P < 0.5

### Construction and verification of the OS nomogram

To develop a survival nomogram, the total dataset was randomly divided into training and validation cohorts using a 7:3 ratio, as shown in Table S4. It can be observed that both datasets have baseline comparability. According to the outcomes of Cox analysis results (Table S5), 3 characteristics (NLR, stage and M) were eventually incorporated into the training cohort survival nomogram development.

**Fig. 3:**
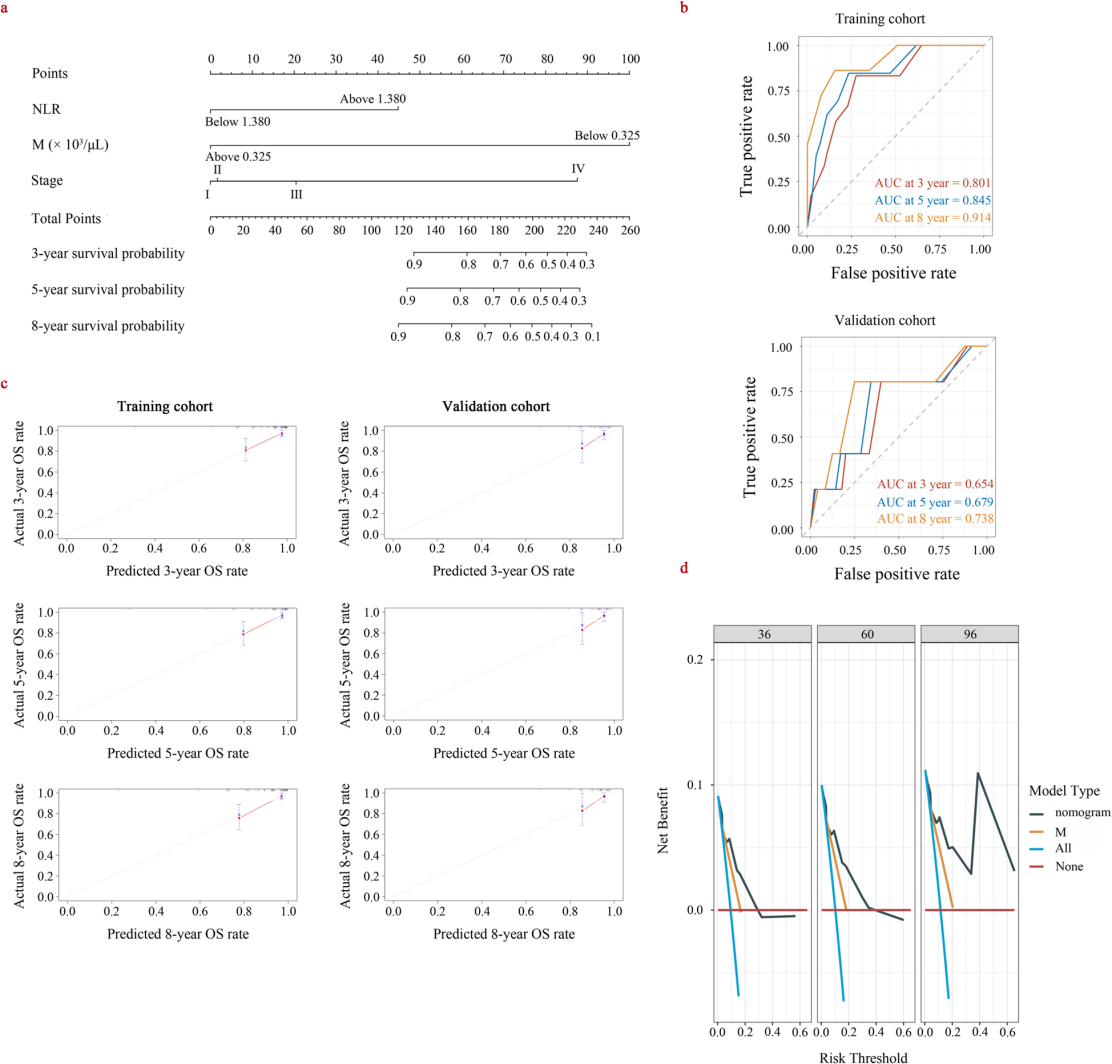
The nomogram of predicting OS in patients with WT and the validation of this model. **a **Survival nomogram for the prediction of 3-year, 5-year, and 8-year OS in WT patients. The patient score for each axis is marked, summing them to obtain a total score. Place the total score on the total points axis and draw a vertical line,so that the 3-, 5-, and 8-year OS rates for WT were determined. **b **Predictive performance of the survival nomogram for Wilms tumor patients assessed by ROC curves. The y-axis represents sensitivity and the x-axis represents specificity for predicting the OS of WT. AUC was the area under the ROC curve. The AUC value closer to 1 indicates higher accuracy of the model. **c **The calibration curve of 3-year, 5-year, and 8-year OS was predicted by the training, validation cohort of WT patients. the diagonal on the chart indicates equal predicted and actual OS rates. The closer the calibration curve is to the diagonal line, the better the predicted OS aligns with the actual OS rate. **d** DCA curves employed for model validation. The x-axis is the threshold probability, and the y-axis is the net benefit. The red line indicates that no patients have died, and the light green line indicates that all patients have died. M absolute monocyte count, OS overall survival, AUC the area under the receiver operating characteristic curve

As illustrated in Fig. 3a, the survival nomogram intuitively predicted the 3-, 5- and 8-year OS rates of WT patients in the training cohort. The C-index of the training and validation cohorts was 0.843 (95%CI 0.765 ~ 0.920) and 0.701 (95%CI 0.439 ~ 0.963), respectively. Specifically, ROC analysis showed that the survival nomogram correctly predicted 3 (AUC = 0.801), 5 (AUC = 0.845) and 8 (AUC = 0.914) year survival for WT patients. (see Fig. 3b). The calibration curve of the survival nomogram is shown in Fig. 3c, which approached the diagonal, indicating good calibration of the survival nomogram in the training cohort [15]. The DCA of the training and the validation cohort indicated that the clinical value of the nomogram is excellent. (Fig. 3d).

## Discussion

In this study, we describe a comprehensive real-world analysis of patients with Wilms tumor over a 30-year period, aiming to identify a convenient prognostic factor based on peripheral blood cells. As seen in the results the 5-year OS of WT was 90.22%, which is consistent with previous research (89%-98%) [16]. Furthermore, the study identified two independent prognostic indicators for WT: stage and absolute monocyte count. These findings can be instrumental in assisting clinicians in early identification of high-risk patients and prompt implementation of advanced treatment strategies.

Stage has been consistently identified as a significant prognostic factor in many tumors. In Wilms tumor, it is well-established that higher stages are associated with worse prognosis [17]. This result was also confirmed in ours study. In this paper, we derived a 5-year OS rate of 68.97% for stage IV patients, which was significantly lower than the overall survival rate (90.22%).

Monocytes, as innate immune cells of the mononuclear phagocyte system, have been recognized as important regulators in cancer development and progression. The present study is the first to report that the absolute pre-treatment peripheral blood monocyte count can be used as a predictor of prognosis in WT, which has not been previously reported. The results of this study indicated that patients with monocyte counts less than 0.325 × 10^3^/μL had lower overall survival and event-free survival rate. The multi-COX model, including traditional factors, further validated that monocytes can be a prognostic risk factor independent of,stage and type. Meanwhile, the construction of the nomogram also demonstrates the predictability of monocytes. Previous research has primarily focused on the function and mechanisms of monocytes within the tumor microenvironment. For instance, Meng et al. reported that a protective effect of monocyte infiltration in the WT tumor microenvironment, while monocyte-derived macrophages had an inhibitory effect [18]. Fiore et al., in their analysis of epithelial and blastemal type WT cells, found that these cells polarized monocytes towards selectively activated macrophages (M2) [19]. Tian et al. suggested that patients with high M2 macrophage densities had shorter OS than those with low densities (log-rank test, *p* = 0.011) [20]. However, the association between monocytes in the peripheral blood and those within the tumor microenvironment remains unclear and requires further in-depth investigation through multicenter and multisubgroup studies.

Recent years, an increasing number of articles have included NLR in the prognostic analysis of tumors. Kunc et al. reported statistically significant results of log-rank analysis of NLR versus overall survival of WT [12]. This result is in line with the results of our paper. As for this variable, it is not significant in multivariate analysis, but the reason why it is significant in the Log-rank test may be due to the influence of confounding factors.

The association of age > 3 years and uFH type with worse prognosis in WT has been reported in several papers [21], but in our article, which was not statistically significant. The possible reason was that the number of cases and positive events were limited, resulting in a seemingly consistent trend in mortality between different age and type groups.

The nomogram was widely applied to develop prognostic predictive models in many diseases. In our study, a nomogram model was constructed based on COX regression. The validation results show that the C-index of the survival nomogram in the training cohort and validation cohort are 0.843 and 0.701, respectively, which indicates the potential prediction performance. Meanwhile, the calibration curve of predicted survival probability is consistent with the actual survival probability. The DAC curve also shows the high utility of the monogram model. However, due to the lack of the enough cases and external verification in this nomogram, we only use it to study the predictability of monoceytes and stage in the study.

This study also has some limitations. Firstly, it is a retrospective study. Many confounding factors may have caused unintentional bias. Secondly, this was a single-center study with a single patient source and the number of cases with positive outcome events was too small. Larger cohorts and prospective studies are expected to validate our models. Third, this study spanned a period of 30 years. Although there was no significant difference in survival between time periods after subgroup analyses (relevant information has been placed in Fig.S3 of the additional file), there was still a potential bias in terms of diagnostic and treatment techniques, patients’ nutritional status, and environment, etc.… In the next step, we hope to conduct a multi-center and large-sample study to analyze the differences in the pre- and post-treatment distribution of monocyte subgroups among the Tumor microenvironment, paraneoplastic and peripheral blood.

## Conclusion

Overall survival and event-free survival were worse in patients with absolute monocyte count below 0.325 × 10^3^/μL and stage IV. For these patients, vigilance regarding their prognosis as well as timely and proactive treatment was required.

## Supplementary Information

Supplementary Material 1.

## Data Availability

The datasets used and/or analysed during the current study are available from the corresponding author on reasonable request.

## Abbreviations

WT: Wilms tumor
OS: Overall survival
EFS: Event-free survival
ROC: Receiver operating characteristiccurve
DCA: Decision curve analysis
AUC: Area under the ROC curve
C-index: Consistency index
NLR: Neutrophil–lymphocyte ratio
CRP: C-reactive protein
LMR: Lymphocyte-to-monocyte ratio
WBC: White blood cell
RBC: Red blood cell
PLT: Platelet count
HB: Hemoglobin count
LB: Lbsolute lymphocyte count
N: Absolute neutrophil count
M: Absolute monocyte count
LB%: Lymphocyte percentage
N%: Neutrophil percentage
M%: M%:Monocyte percentage
PLR: Platelet-lymphocyte ratio
FH: Favorable histology
uFH: Unfavorable histology
