# Supplementary material for: Predicting the prognosis of Wilms tumor by peripheral blood cells: a real-world study of more than 30 years
Source: Ital J Pediatr. 2024 Nov 14;50:245. doi: 10.1186/s13052-024-01805-8 (PMC11566747; doi:10.1186/s13052-024-01805-8)
Supplement: Supplementary file 1 — Supplementary Material 1. [file 13052_2024_1805_MOESM1_ESM.pdf]

Table S1 Blood cells compared between patients with WT and non-WT

Variables	non-WT (n=60)	WT (n=184)	*P*
PLR	63.82 (51.62, 91.93)	94.63 (62.20, 136.27)	0.000**
NLR	0.71 (0.46, 0.94)	1.38 (0.78, 2.11)	0.000**
WBC (× 10^3/μL)	8.25(6.60,9.20)	9.27(7.30,11.80)	0.003**
PLT (× 10^3/μL)	299.00(259.80,336.00)	326.50(266.30,436.80)	0.006**
RBC (× 10^6/μL)	4.51(4.30,4.80)	4.27(3.90,4.60)	0.000**
HB (g/L)	124.00(118.00,130.80)	108.00(99.30,119.00)	0.000**
LB (× 10^3/μL)	4.34(3.30,5.40)	3.57(2.60,5.10)	0.011*
N (× 10^3/μL)	3.12(2.10,3.70)	4.78(3.40,6.40)	0.000**
M (× 10^3/μL)	0.39(0.30,0.50)	0.33(0.20,0.40)	0.005**
Lb%	0.54(0.49,0.63)	0.41(0.30,0.53)	0.000**
N%	0.38(0.30,0.43)	0.54(0.41,0.62)	0.000**
M%	0.04(0.03,0.05)	0.03(0.03,0.04)	0.000**
Age (mon)	27.00(20.50,31.80)	30.50(16.00,53.80)	0.344
Gender			0.608
Males	30(50.00)	85(46.20)	
Females	30(50.00)	99(53.80)	

The categorical data were presented as n (%) and the continuous variable data were expressed as median (25th percentile, 75th percentile). * P<0.5, ** P<0.01, *** P<0.001, WBC white blood cell count, PLT platelet count, RBC red blood cell count, HB hemoglobin, LB absolute lymphocyte count, N absolute neutrophil count, M absolute monocyte count, LB% lymphocyte percentage, N% neutrophil percentage, M% monocyte percentage, PLR platelet-lymphocyte ratio, NLR neutrophil-lymphocyte ratio, P P-value, n number

Table S2 Multifactorial COX regression of peripheral blood cells and OS in total data

Variables		*P*	HR (95%CI)
NLR			
	Below 1.380		Ref
	Above 1.380	0.053	2.89 (0.99 ~ 8.43)
M (× 10^3/μL)			
	Below 0.325		Ref

	Above 0.325	0.004*	0.22 (0.08 ~ 0.62)
Stage			
	I		Ref
	II	0.870	1.16 (0.19 ~ 6.99)
	III	0.987	1.01 (0.18 ~ 5.77)
	IV	0.010*	7.89 (1.65 ~ 37.77)

* P<0.5, ** P<0.01, *** P<0.001, HR Hazard Ratio, CI Confidence Interval, Ref reference

Table S3 Multifactorial COX regression of peripheral blood cells and EFS in total data

Variables		*p*	HR (95%CI)
LB (× 10^3/μL)			
	Below 3.570		Ref
	Above 3.570	0.727	0.82(0.27 ~ 2.52)
M (× 10^3/μL)			
	Below 0.325		Ref
	Above 0.325	0.036*	0.44(0.20 ~ 0.95)
PLR			
	Below 94.632		Ref
	Above 94.632	0.697	1.24(0.42 ~ 3.68)
Age (mon)			
	≤3years		Ref
	＞3years	0.230	1.68(0.72 ~ 3.89)
Stage			
	I		Ref
	II	0.923	1.05(0.36 ~ 3.07)
	III	0.422	0.62(0.19 ~ 1.99)
	IV	0.012*	3.72(1.33 ~ 10.41)

* P<0.5, ** P<0.01, *** P<0.001, HR Hazard Ratio, CI Confidence Interval, Ref reference

Table S4 Clinicopathological characteristics of training cohort and validation cohort

Variables		Training cohort (n = 128)	Validation cohort (n = 56)	P
PLR				0.749
	Below 94.632	65 (50.78)	27 (48.21)	
	Above 94.632	63 (49.22)	29 (51.79)	
NLR				0.522
	Below 1.380	66 (51.56)	26 (46.43)	
	Above 1.380	62 (48.44)	30 (53.57)	
WBC (× $10^3/\mu L$)				1.000
	Below 9.270	64 (50.00)	28 (50.00)	
	Above 9.270	64 (50.00)	28 (50.00)	
PLT (× $10^3/\mu L$)				0.336
	Below 326.500	61 (47.66)	31 (55.36)	
	Above 326.500	67 (52.34)	25 (44.64)	
RBC (× $10^6/\mu L$)				0.443
	Below 4.270	65 (50.78)	25 (44.64)	
	Above 4.270	63 (49.22)	31 (55.36)	
HB (g/L)				0.706
	Below 108.000	61 (47.66)	25 (44.64)	
	Above 108.000	67 (52.34)	31 (55.36)	
LB (× $10^3/\mu L$)				0.089
	Below 3.570	58 (45.31)	33 (58.93)	

	Above 3.570	70 (54.69)	23 (41.07)	
N (× 10^3/μL)				0.336
	Below 4.780	67 (52.34)	25 (44.64)	
	Above 4.780	61 (47.66)	31 (55.36)	
M (× 10^3/μL)				0.200
	Below 0.325	60 (46.88)	32 (57.14)	
	Above 0.325	68 (53.12)	24 (42.86)	
LB%				0.676
	Below 0.410	62 (48.44)	29 (51.79)	
	Above 0.410	66 (51.56)	27 (48.21)	
N%				0.522
	Below 0.535	66 (51.56)	26 (46.43)	
	Above 0.535	62 (48.44)	30 (53.57)	
M%				0.083
	Below 0.030	26 (20.31)	18 (32.14)	
	Above 0.030	102 (79.69)	38 (67.86)	
Age (mon)				0.095
	≤3years	83 (64.84)	29 (51.79)	
	＞3years	45 (35.16)	27 (48.21)	
Gender				0.716
	Females	58 (45.31)	27 (48.21)	
	Males	70 (54.69)	29 (51.79)	

Stage				0.195
	I	34 (26.56)	7 (12.50)	
	II	35 (27.34)	18 (32.14)	
	III	41 (32.03)	20 (35.71)	
	IV	18 (14.06)	11 (19.64)	
Type				0.255
	FH	115 (89.84)	47 (83.93)	
	uFH	13 (10.16)	9 (16.07)	
Laterality				0.567
	Left	63 (49.22)	25 (44.64)	
	Right	65 (50.78)	31 (55.36)	

Data are presented as n(%). * P<0.5, ** P<0.01, *** P<0.001, WBC white blood cell count, PLT platelet count, RBC red blood cell count, HB hemoglobin, LB absolute lymphocyte count, N absolute neutrophil count, M absolute monocyte count, LB% lymphocyte percentage, N% neutrophil percentage, M% monocyte percentage, PLR platelet-lymphocyte ratio, NLR neutrophil-lymphocyte ratio, P P-value, n number, FH favorable histology, uFH unfavorable histology

Table S5 Univariate and multivariate COX regression analyses of OS in training cohort

Variables	Univariate			Multivariate		
	HR	95%CI	P	HR	95%CI	P
PLR						

	Below 94.632	Ref					
	Above 94.632	4.15	1.16 - 14.88	0.029*			
NLR							
	Below 1.380	Ref			Ref		
	Above 1.380	2.81	0.88 - 8.98	0.080	2.77	0.83 - 9.18	0.096
WBC (× 10^3/μL)							
	Below 9.270	Ref					
	Above 9.270	1.89	0.63 - 5.64	0.255			
PLT (× 10^3/μL)							
	Below 326.500	Ref					
	Above 326.500	1.47	0.51 - 4.25	0.473			
RBC (× 10^6/μL)							
	Below 4.270	Ref					
	Above 4.270	1.33	0.46 - 3.82	0.602			
HB (g/L)							
	Below 108.000	Ref					

	Above 108.000	0.82	0.28 - 2.36	0.711			
LB (× $10^3/\mu L$)							
	Below 3.570	Ref					
	Above 3.570	3.36	1.05 - 10.71	0.041*			
N (× $10^3/\mu L$)							
	Below 4.780	Ref					
	Above 4.780	0.82	0.28 - 2.37	0.714			
M (× $10^3/\mu L$)							
	Below 0.325	Ref			Ref		
	Above 0.325	0.14	0.03 - 0.62	0.010*	0.10	0.02 - 0.46	0.003*
LB%							
	Below 0.410	Ref					
	Above 0.410	2.83	0.89 - 9.04	0.078			
N%							
	Below 0.535	Ref					
	Above 0.535	1.99	0.67 - 5.94	0.217			
M%							
	Below 0.030	Ref					
	Above 0.030	0.45	0.15 - 1.35	0.153			
Age (mon)							

	≤3years	Ref					
	＞3years	3.69	1.24 - 11.03	0.019*			
Gender							
	Females	Ref					
	Males	1.20	0.42 - 3.42	0.734			
Stage							
	I	Ref			Ref		
	II	0.97	0.14 - 6.93	0.980	0.96	0.13 - 6.92	0.970
	III	1.74	0.32 - 9.52	0.521	1.53	0.28 - 8.43	0.624
	IV	6.90	1.39 - 34.20	0.018*	7.04	1.41 - 35.29	0.018*
Type							
	FH	Ref					
	uFH	1.60	0.36 - 7.16	0.540			
Laterality							
	Left	Ref					
	Right	1.92	0.64 - 5.74	0.241			

* P<0.5, ** P<0.01, *** P<0.001, WBC white blood cell count, PLT platelet count, RBC red blood cell count, HB hemoglobin, LB absolute lymphocyte count, N absolute neutrophil count, M absolute monocyte count, LB% lymphocyte percentage, N% neutrophil percentage, M% monocyte percentage, PLR platelet-lymphocyte ratio, NLR neutrophil-lymphocyte ratio, P P-value, n number, FH favorable histology, uFH unfavorable histology, Ref reference

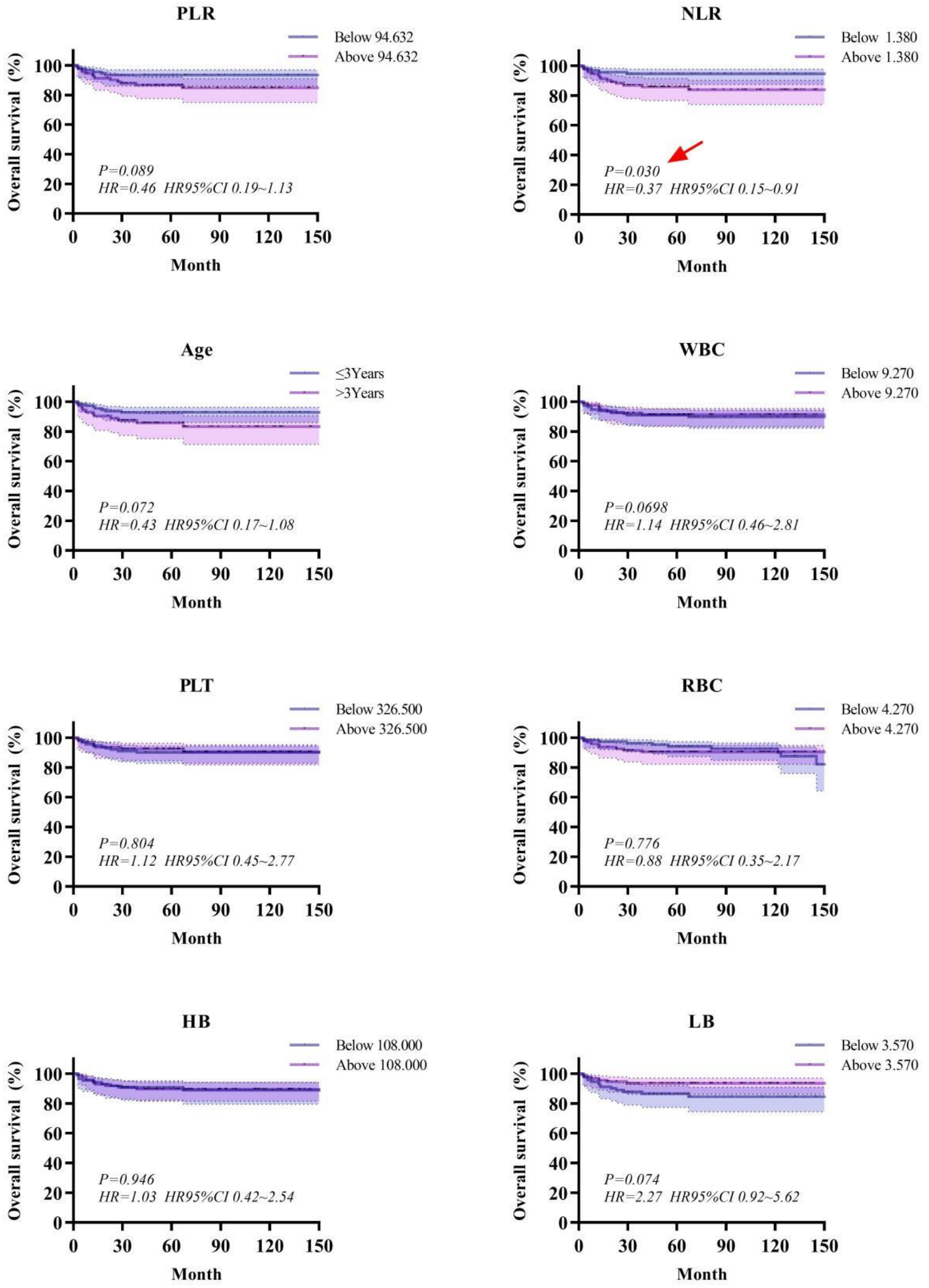
PLR
Below 94.632
Above 94.632
Overall survival (%)
P=0.089
HR=0.46 HR95%CI 0.19~1.13
Month
NLR
Below 1.380
Above 1.380
P=0.030
HR=0.37 HR95%CI 0.15~0.91
Age
≤3Years
>3Years
P=0.072
HR=0.43 HR95%CI 0.17~1.08
WBC
Below 9.270
Above 9.270
P=0.0698
HR=1.14 HR95%CI 0.46~2.81
PLT
Below 326.500
Above 326.500
P=0.804
HR=1.12 HR95%CI 0.45~2.77
RBC
Below 4.270
Above 4.270
P=0.776
HR=0.88 HR95%CI 0.35~2.17
HB
Below 108.000
Above 108.000
P=0.946
HR=1.03 HR95%CI 0.42~2.54
LB
Below 3.570
Above 3.570
P=0.074
HR=2.27 HR95%CI 0.92~5.62

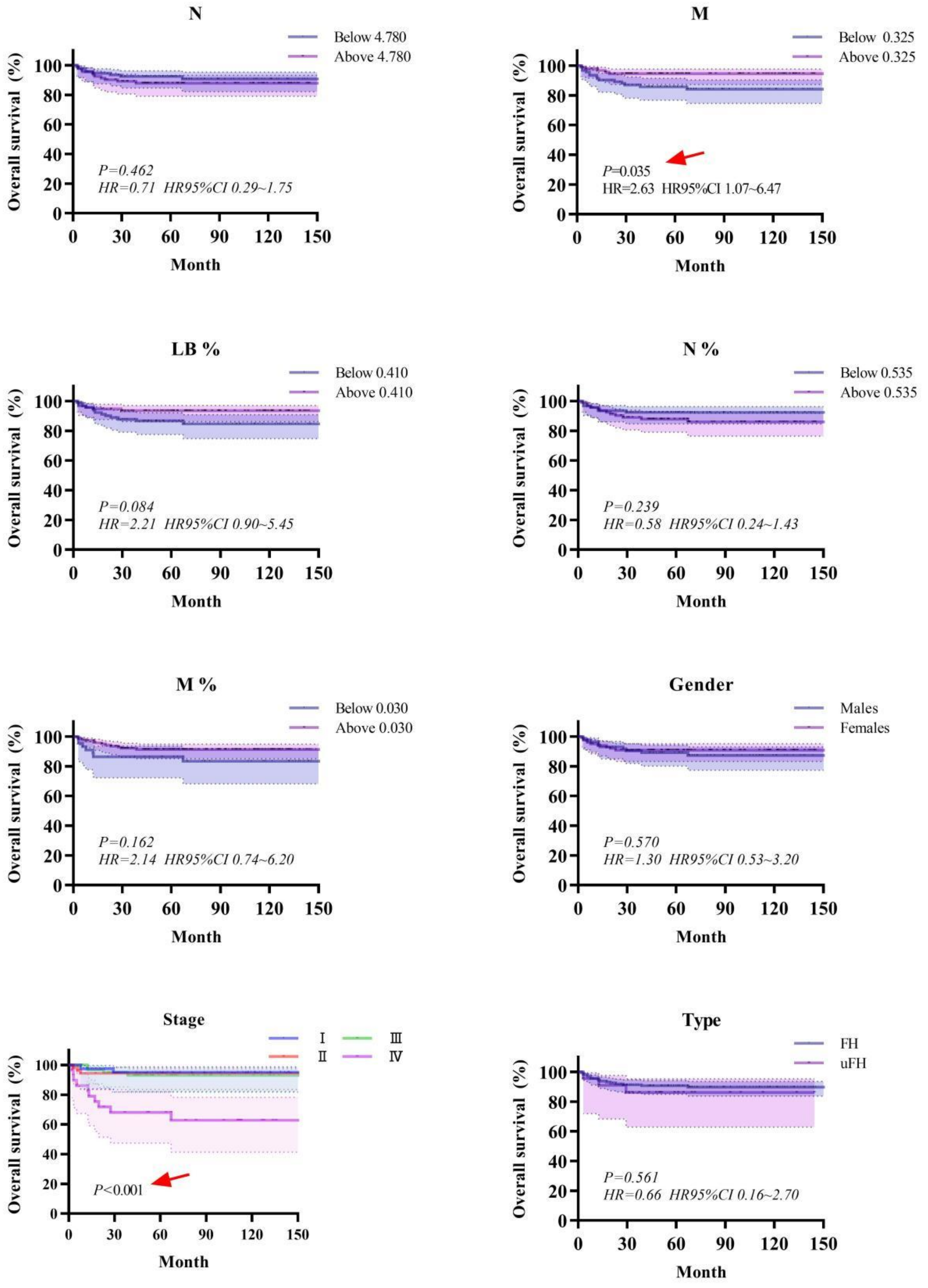

Fig.S1 K-M curve of OS in WT. WBC white blood cell count, PLT platelet count, RBC red blood cell count, HB hemoglobin, LB absolute lymphocyte count, N absolute neutrophil count, M absolute monocyte count, LB% lymphocyte percentage, N% neutrophil percentage, M% monocyte percentage, PLR platelet-lymphocyte ratio, NLR neutrophil-lymphocyte ratio, P P-value, n number, OS overall survival

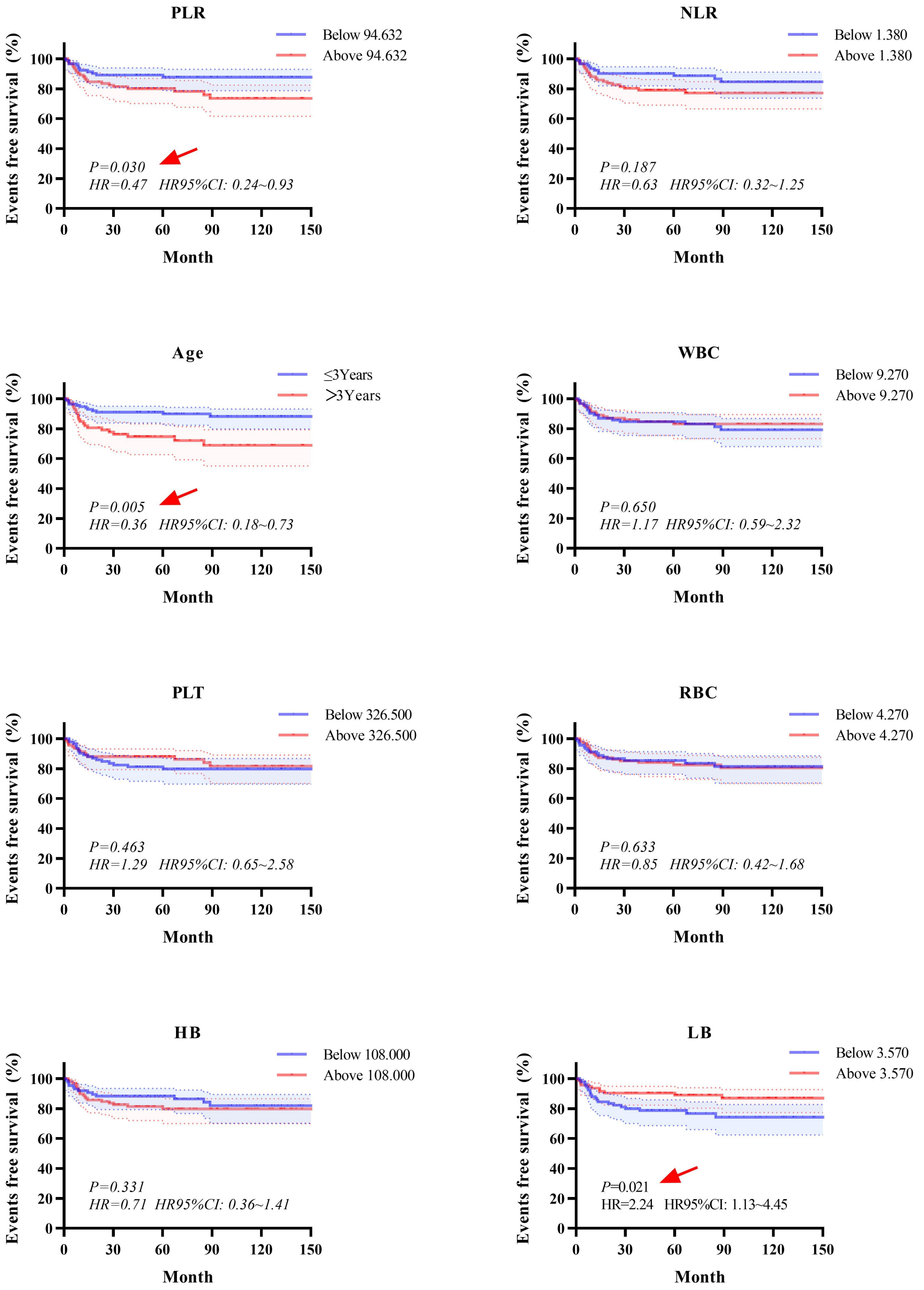
PLR
Below 94.632
Above 94.632
P=0.030
HR=0.47 HR95%CI: 0.24~0.93
NLR
Below 1.380
Above 1.380
P=0.187
HR=0.63 HR95%CI: 0.32~1.25
Age
≤3Years
>3Years
P=0.005
HR=0.36 HR95%CI: 0.18~0.73
WBC
Below 9.270
Above 9.270
P=0.650
HR=1.17 HR95%CI: 0.59~2.32
PLT
Below 326.500
Above 326.500
P=0.463
HR=1.29 HR95%CI: 0.65~2.58
RBC
Below 4.270
Above 4.270
P=0.633
HR=0.85 HR95%CI: 0.42~1.68
HB
Below 108.000
Above 108.000
P=0.331
HR=0.71 HR95%CI: 0.36~1.41
LB
Below 3.570
Above 3.570
P=0.021
HR=2.24 HR95%CI: 1.13~4.45
Events free survival (%)
Month
0
30
60
90
120
150
0
20
40
60
80
100

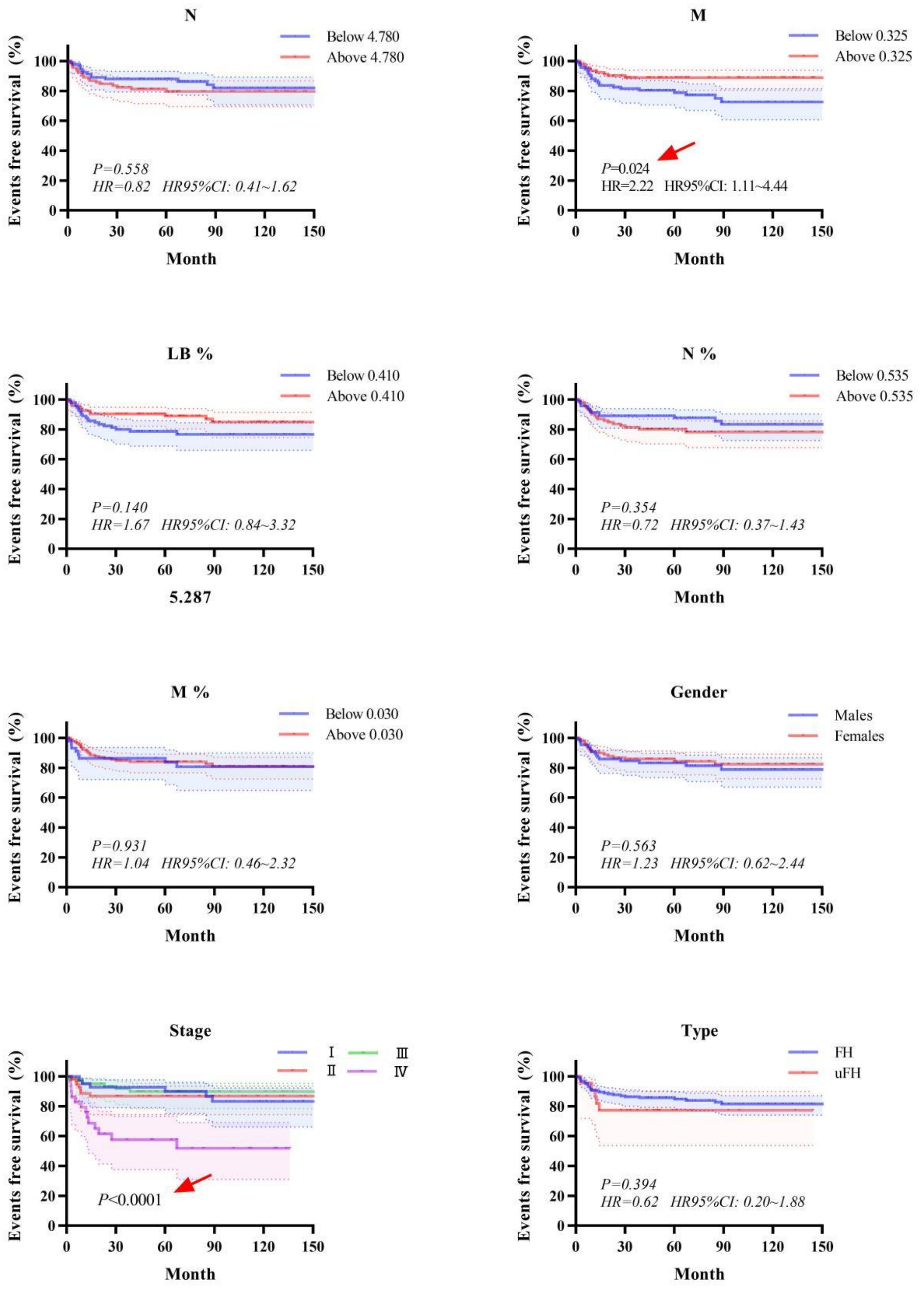

Fig.S2 K-M curve of EFS in WT. WBC white blood cell count, PLT platelet count, RBC red blood cell count, HB hemoglobin, LB absolute lymphocyte count, N absolute neutrophil count, M absolute monocyte count, LB% lymphocyte percentage, N% neutrophil percentage, M% monocyte percentage, PLR platelet-lymphocyte ratio, NLR neutrophil-lymphocyte ratio, P P-value, n number, OS overall survival

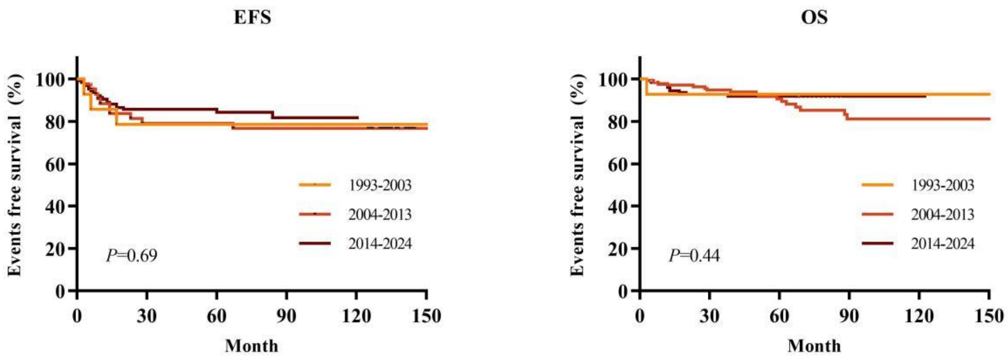

Fig.S3 The K-M curves of OS and EFS at different time points. This graph evident that there is no significant difference in EFS and OS among patients with WT treated at our hospital across different time intervals. P P-value, n number, OS overall survival, EFS event-free survival
